# Association of chemokine receptor gene (*CCR2-CCR5*) haplotypes with acquisition and control of HIV-1 infection in Zambians

**DOI:** 10.1186/1742-4690-8-22

**Published:** 2011-03-23

**Authors:** Rakhi Malhotra, Liangyuan Hu, Wei Song, Ilene Brill, Joseph Mulenga, Susan Allen, Eric Hunter, Sadeep Shrestha, Jianming Tang, Richard A Kaslow

**Affiliations:** 1 Department of Epidemiology University of Alabama at Birmingham (UAB), Birmingham, AL, USA; 2 Department of Medicine, University of Alabama at Birmingham (UAB), Birmingham, AL, USA; 3 Rwanda-Zambia HIV-1 Research Group, Lusaka, Zambia; 4 Emory University, Atlanta, GA, USA

## Abstract

**Background:**

Polymorphisms in chemokine (C-C motif) receptors 2 and 5 genes (*CCR2 *and *CCR5*) have been associated with HIV-1 infection and disease progression. We investigated the impact of *CCR2-CCR5 *haplotypes on HIV-1 viral load (VL) and heterosexual transmission in an African cohort. Between 1995 and 2006, cohabiting Zambian couples discordant for HIV-1 (index seropositive and HIV-1 exposed seronegative {HESN}) were monitored prospectively to determine the role of host genetic factors in HIV-1 control and heterosexual transmission. Genotyping for eight *CCR2 *and *CCR5 *variants resolved nine previously recognized haplotypes. By regression and survival analytic techniques, controlling for non-genetic factors, we estimated the effects of these haplotypic variants on a) index partner VL, b) seroconverter VL, c) HIV-1 transmission by index partners, d) HIV-1 acquisition by HESN partners.

**Results:**

Among 567 couples, 240 virologically linked transmission events had occurred through 2006. HHF*2 homozygosity was associated with significantly lower VL in seroconverters (mean beta = -0.58, log_10 _*P *= 0.027) and the HHD/HHE diplotype was associated with significantly higher VL in the seroconverters (mean beta = 0.54, log_10 _*P *= 0.014) adjusted for age and gender in multivariable model. HHD/HHE was associated with more rapid acquisition of infection by the HESNs (HR = 2.0, 95% CI = 1.20-3.43, *P *= 0.008), after adjustments for index partner VL and the presence of genital ulcer or inflammation in either partner in Cox multivariable models. The HHD/HHE effect was stronger in exposed females (HR = 2.1, 95% CI = 1.14-3.95, *P *= 0.018).

**Conclusions:**

Among Zambian discordant couples, HIV-1 coreceptor gene haplotypes and diplotypes appear to modulate HIV-1 VL in seroconverters and alter the rate of HIV-1 acquisition by HESNs. These associations replicate or resemble findings reported in other African and European populations.

## Background

Sub-Saharan Africa is home to about 10% of the world's population but bears nearly 64% of all HIV-1 infections [1], with most HIV-1 transmission occurring heterosexually. In Zambia, about one in five cohabiting couples involves an HIV-1 seropositive (index) and a seronegative (exposed) partner; these serodiscordant couples are at high risk of heterosexual transmission, with an estimated rate of eight transmission events per 100 person-years of follow-up [2].

The rate of within-couple heterosexual HIV-1 transmission is highly variable, and a number of viral, host and environmental factors may modify transmission (infectiousness), acquisition (susceptibility) or both [3]. Donor HIV-1 viral load (VL), age, sex, history of sexually transmitted infection (STI), unprotected sex, and possible HIV-1 subtype are among the major factors implicated [4,5]. In southern Africa, unusual biological features of the predominant C subtype of HIV-1 [5] and absence of the human CC chemokine receptor 5 gene (*CCR5*) 32-bp deletion (Δ32) as a resistance factor may contribute to relatively high transmission rate.

The recognition that Caucasians who are homozygous for CCR5-Δ32 are highly resistant to HIV-1 infection was a landmark finding in research on HIV-1 transmission [6-9]. It stimulated a concerted effort to elucidate the impact of other genetic variations in *CCR5 *and the adjacent gene *CCR2 *on HIV-1 transmission and disease progression [10-12]. Research on the association of these variants with transmission has been largely cross-sectional or retrospective; the few prospective studies have focused on vertical (mother-to-child) transmission [13] and on HIV-1 exposed seronegatives (HESNs), in part because of the difficulty in enrolling and following HIV-1-discordant couples.

In Zambia, thousands of cohabiting and HIV-1 discordant couples have been offered voluntary counseling and testing (VCT) services since 1995 [2], and some of them have been followed for more than 10 years [14]. Despite counseling and behavioral interventions, the rate of HIV-1 transmission among these couples has remained high [15]. This circumstance permitted us to investigate the association of polymorphism in *CCR2 *and *CCR5 *with heterosexual transmission of phylogenetically related [16] HIV-1 within discordant partners.

The major published studies [11,13,17-20] examining the effects of *CCR2 *and *CCR5 *SNPs/haplotypes/diplotypes on HIV-1 infection or disease progression have shown a wide spectrum of effects in various populations (See Additional File 1; Table S1). We systematically tested hypotheses on these as well as other markers that occurred frequently enough in our population to permit meaningful inferences, especially in confirmation of earlier findings.

## Results

### General characteristics of Zambian couples with linked HIV-1 viruses

During the study period 567 couples were eligible for analysis. Linked transmission occurred in 240 of the 567; (Table 1). nearly all (> 95%) of the HIV-1 sequences from these transmission pairs corresponded to viral subtype C (HIV-1C) [16]. Male-to-female transmission accounted for nearly three-fifths of the incident infections (Table 1). The younger age of exposed women and, to a lesser extent, exposed men was associated with seroconversion. Certain non-genetic characteristics of the partners were also independently associated with increased transmission: genital ulcers or genital inflammation in any partner (HR = 3.62, 95% CI: 2.65-4.93, *P *< 0.0001) and high VL in the index partner (HR = 1.59, 95% CI: 1.32-1.91, *P *< 0.0001). These factors were retained in subsequent models that tested the impact of genetic markers.

**Table 1:** Demographic, epidemiologic and virologic characteristics of the HIV-1 nontransmission and transmission serodiscordant Zambian couples

	Nontransmission couples	Transmission couples	P
**Characteristic**			

Number of couples	327	240	
Male/Female (index partner)	148/179	147/93	0.0002
Age of partners (yrs)			
Index	31.5 ± 7.9	30.6 ± 7.8	0.170
Exposed	32.0 ± 8.3	28.6 ± 7.3	< 0.0001
Follow-up time (median [IQR], months)	31.5 [17.0-56.1]	17.7 [8.8-36.2]	< 0.0001
Male circumcised			
Index	8.8%	9.0%	0.970
Exposed	19.5%	9.9%	0.053
Genital ulcers			
Index	12.8%	36.6%	< 0.0001
Exposed	4.1%	26.3%	< 0.0001
Genital inflammation ^a^			
Index	10.3%	26.3%	< 0.0001
Exposed	6.6%	29.1%	< 0.0001
Any sexually transmitted disease			
Index	20.7%	50.2%	< 0.0001
Exposed	10.0%	46.0%	< 0.0001
HIV-1 RNA level (log_10_) in index partner	4.47 ± 0.90	4.96 ± 0.70	< 0.0001
HIV-1 RNA level (log_10_) in seroconverted partner		4.50 ± 0.80	NA

^a ^In the 3-6 months before HIV-1 transmission (transmission couples) or latest follow-up visit (nontransmission couples).

### Distribution of *CCR2-CCR5 *haplotypes in Zambian couples

Eight *CCR2-CCR5 *haplotypes were observed in the frequency distribution shown in Table 2. Nearly 50% of all haplotypes were HHA or HHF*2. Haplotype HHB was rarely seen, and the Δ32-containing haplotype HHG*2 was not observed at all. The most common genotypes (diplotypes) were HHA/HHF*2, HHA/HHD, HHA/HHA, HHA/HHE, HHD/HHF*2, and HHE/HHF*2 (See Additional File 2; Table S2). The overall distribution of *CCR2-CCR5 *haplotypes did not conform to HWE (Table 2). After stratification of the cohort into transmission and nontransmission index partners, seroconverters, and exposed uninfected partners, the haplotype distribution deviated significantly from HWE in all three seropositive groups, but not in the HESNs.

**Table 2:** Frequencies of *CCR2*-*CCR5 *polymorphisms among HIV-1 serodiscordant couples, index partners, and HIV-1 exposed seronegative partners

			HESN^a ^partners		
			
	All (N = 1134)	Index partners (N = 567)	All (N = 567)	Seroconverters (N = 240)	Uninfected (N = 327)
Haplotype	N (%)	N (%)	N (%)	N (%)	N (%)

HHA	601 (26.5)	305 (26.9)	296 (26.1)	133 (27.7)	163 (24.9)
HHB	44 (1.9)	25 (2.2)	19 (1.7)	8 (1.7)	11 (1.7)
HHC	178 (7.9)	69 (6.1)	109 (9.6)	42 (8.8)	67 (10.2)
HHD	370 (16.3	190 (16.8)	180 (15.9)	75 (15.6)	105 (16.1)
HHE	310 (13.7)	138 (12.2)	172 (15.2)	61 (12.7)	111 (17.0)
HHF*1	128 (5.6)	76 (6.7)	52 (4.6)	24 (5.0)	28 (4.3)
HHF*2	480 (21.2)	259 (22.8)	221 (19.5)	99 (20.6)	122 (18.7)
HHG*1	157 (6.9)	72 (6.4)	85 (7.5)	38 (7.9)	47 (7.2)

HWE:P^b^	0.0001	0.0001	0.267	0.024	0.160

^a ^HESN = HIV-1 exposed seronegative

^b ^P values for tests of Hardy-Weinberg equilibrium in each of the patient groups.

### *CCR2-CCR5 *determinants of VL

Although HHA and HHC have previously shown protective effects in the form of associations with lower VL in a mixed population[21], we did not observe such an effect on VL in Zambians with either haplotype overall or with any specific diplotypes containing either of them.

In prior studies, HHF*2 has shown somewhat inconsistent associations with VL and disease control [11,17,19,20,22,23]. In our Zambian study population HHF*2 showed a weak association with lower VL in both index partners (β = -0.21, log_10 _*P *= 0.024) and seroconverters (β = -0.10, log_10 _*P *= 0.089). When the index partners and seroconverters were stratified by HHF*2 genotype, a stronger association in the latter group was largely attributable to HHF*2 homozygosity (β = -0.70, log_10 _*P *= 0.007) (Table 3).

**Table 3:** The impact of *CCR2-CCR5 *haplotypes on HIV-1 viral load in Zambian index partners and seroconverters

	Index partners			Recent seroconverters		
	(N = 567)			(N = 240)		
**Viral Load Table for CCR5 haplotype/diplotype**						

Haplotype/diplotype	N	β ± SE^a^	P*	N	β ± SE^a^	P*
HHF*2	232	-0.21 ± 0.09	0.024	89	-0.10 ± 0.58	0.089
HHF*2/HHF*2	27	-0.08 ± 0.16	0.625	10	-0.70 ± 0.26	0.007
HHD (All)	164	-0.12 ± 0.06	0.052	69	0.24 ± 0.10	0.021
HHE (All)	131	0.13 ± 0.08	0.096	61	0.12 ± 0.12	0.339
HHD/HHE	18	-0.22 ± 0.20	0.270	16	0.49 ± 0.21	0.020
HHD/X (No HHE)	146	-0.11 ± 0.08	0.157	53	0.14 ± 0.13	0.284
HHE/X (No HHD)	113	0.19 ± 0.09	0.026	45	-0.06 ± 0.13	0.673

Multivariable Model for Interaction**						

Haplotype/diplotype	N	β ± SE^a^	P*	N	β ± SE^a^	P*
HHD/X (No HHE or HHF*2)	109	-0.16 ± 0.09	0.560	37	0.24 ± 0.16	0.118
HHE/X (No HHD or HHF*2)	69	0.36 ± 0.11	0.002	31	0.10 ± 0.16	0.556
HHD/HHE	18	-0.16 ± 0.20	0.444	16	0.54 ± 0.22	0.014
HHF*2/HHF*2	27	-0.02 ± 0.16	0.918	10	-0.58 ± 0.26	0.027
HHF2/X (No HHD, HHE or HHF*2)	124	0.05 ± 0.09	0.602	49	0.10 ± 0.14	0.496

^a ^SE, standard error of the β estimate according to linear regression models.

* P value adjusted for the sex and age at VL for all the individuals.

** Individuals with HHD/HHF*2, HHE/HHF*2 diplotypes are in the reference group in the multivariable model for interaction.

Both HHD and HHE have been associated with higher VL in several studies [11,13,18,24,25]. In our Zambian cohort, dominant models, including each haplotype plus non-genetic factors analyzed by GLM, indicated that HHD was associated with higher VL (β = 0.24, log_10 _*P *= 0.021) in the seroconverters, but a modest effect in the opposite direction was observed in index partners. HHE showed a trend toward association with higher VL in index partners and a similar non-significant association with higher VL in seroconverters adjusted for age and gender (Table 3). Because this pattern of association could be explained by combinations of haplotypes carried, we explored the effect of diplotypes further. Among all diplotypes of frequency > 0.05, HHD/HHE showed the strongest association with higher VL (β = 0.49, log_10 _*P *= 0.02)

We next constructed a multivariable model with all the haplotypes and diplotypes that showed a trend toward association (log_10 _*P *< 0.10) with higher or lower VL in either index partners or seroconverters to test their independent influences on VL (Table 3). In this model, by including uninformative diplotypes (HHD/HHF*2 and HHE/HHF*2) in the reference group, each diplotype implicated could be tested independently of the others. In the index partners, HHE/X shows a strong association with higher VL. In seroconverters the HHD/HHE and HHF*2/HHF*2 diplotypes remained significantly and independently associated with VL after controlling for individual haplotype effects (Table 3).

### *CCR2-CCR5 *determinants of transmission from index partners and of seroconversion in HESNs

**Figure 1:**
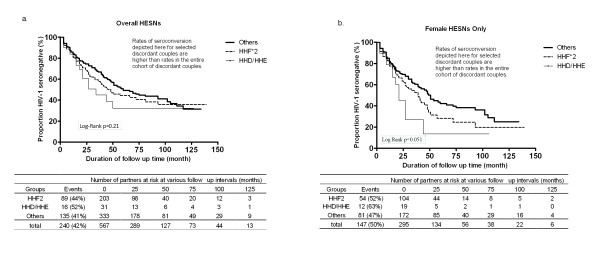
Association of two *CCR2-CCR5 *diplotype (HHD/HHE and HHF*2) with time to HIV-1 acquisition among initially seronegative partners of HIV-1 discordant Zambian couples. Analysis was based on 567 initially seronegative HESNs (panel a) or 295 seronegative female HESNs only (panel b). Vertical lines on each Kaplan-Meier curve represent subjects censored at the last follow up visit (before December 2006).

The few studies that have attempted to assess the role of the receptor polymorphism in transmission and susceptibility have shown rather diverse associations of common *CCR5 *haplotypes, without any discernible pattern (See Additional File 1, Table S1). No SNP or haplotype carried by Zambian index partners was significantly associated with transmission (data not shown). In the survival analysis, HESNs with the HHD/HHE diplotypes showed significantly more rapid seroconversion than HESNs with other haplotypes (Table 4 and Figure 1a) after adjustments for index partner VL and the presence of genital ulcer or inflammation in either partner. Although HHF*2 did not show statistically significant association with faster HIV-1 acquisition, we assigned it to a separate stratum in the Kaplan-Meier plot because aggregating it in the reference group would have given the appearance of a weaker HHD/HHE effect (Multivariable Cox model HR = 2.0, 95% CI = 1.20-3.43, *P *= 0.008). Stratification by gender revealed a stronger impact of HHD/HHE on HESN women than men (Table 4 and Figure 1b) (Multivariable Cox model HR = 2.1, 95% CI = 1.14-3.95, *P *= 0.018).

**Table 4:** Proportional hazards analysis of the effect of *CCR2-CCR5 *haplotype or diplotype on HIV-1 acquisition.

	Overall				Male-to-Female				Female-to-Male			
	(567 couples)				(295 couples)				(272 couples)			
*Cox model for individual CCR2-CCR5 haplotype or diplotype*.												

**Haplotype/diplotype**	N*	HR	95% CI	P**	N*	**HR**	**95% CI**	P**	N*	HR	95% CI	P**

HHF*2	203	1.1	0.85-1.46	0.417	**104**	**1.1**	**0.77-1.56**	**0.61**	99	1.2	0.75-1.75	0.531
**HHD**	167	1.0	0.74-1.32	0.983	**94**	**0.9**	**0.59-1.23**	**0.385**	73	1.2	0.75-1.92	0.442
**HHE**	158	1.1	0.82-1.51	0.495	**85**	**1.3**	**0.86-1.87**	**0.234**	73	0.9	0.57-1.53	0.781
**HHD/HHE**	31	1.9	1.14-3.16	0.015	**19**	**2.0**	**1.08-3.57**	**0.028**	12	1.7	0.60-4.66	0.321

Multivariable model for CCR2-CCR5 HHD/HHE diplotype and HHF*2 haplotype.												

**Genetic factors**	N*	HR	95% CI	P**	N*	**HR**	**95% CI**	P**	N*	HR	95% CI	P**
**HHD/HHE**	31	2.0	1.20-3.43	0.008	**19**	**2.1**	**1.14-3.95**	**0.018**	12	1.8	0.64-5.08	0.267
HHF*2	203	1.2	0.90-1.57	0.222	**104**	**1.2**	**0.83-1.72**	**0.337**	99	1.2	0.77-1.82	0.439
**Any genital ulcer or inflammation**	299	3.6	2.65-4.93	< .0001	**162**	**3.0**	**2.04-4.51**	**< .0001**	137	4.6	2.79-7.65	< .0001
**Donor VL (per 1.0 log_10 _unit)**	523	1.6	1.32-1.91	< .0001	**263**	**1.3**	**1.03-1.74**	**0.028**	260	1.8	1.35-2.47	< .0001

*N represents the number of HESNs with each genotype.

**P values adjusted for genital ulcer, genital inflammation in either partner, and index partner log_10 _VL.

## Discussion

Many investigations into genetic determinants of HIV/AIDS have evaluated the effects of Δ32, selected SNPs, and haplotypes across *CCR2*-*CCR5 *on disease progression in a variety of infected populations. Studies of these markers as determinants of acquisition have usually been conducted in pairs of mothers and infants or in exposed men of European ancestry whose male sexual contacts are largely unknown [13,17,25-27]. Our relatively large prospective study of heterosexual discordant African couples has produced further evidence for involvement of variants in these genes in both control and occurrence of HIV-1 infection.

HHE was associated with slightly higher VL than was seen with other haplotypes, a finding consistent with observations in a number of other studies on different ethnic groups and various modes of transmission [11,17,28,29]. Further confirmation of the effect of HHE highlights its potential impact on clinical HIV-1 disease control in diverse populations, in contrast to that of the protective Δ32 variant whose distribution is confined to individuals of European ancestry. We detected an association of homozygous HHF*2 (containing CCR2-64I) with lower VL in recent seroconverters but found less certain effects of heterozygous HHF*2. This finding is consistent with previous reports [11,17,19,20,22]. Although an early meta-analysis persuasively documented modest protection by the 64I allele against progression of HIV-1 subtype B infection [22], results in subsequent studies have been less consistent--showing association with slow progression either among Europeans, but not African-Americans [19,20] or among African-Americans but not Europeans [23,29]. For populations with subtype C infection, however, no previous study is available as a basis for comparison.

As for the influence of *CCR2-CCR5 *alleles or haplotypes on transmission and acquisition of infection, the highly significant deviation of the distribution of haplotypes from HWE among the index, but not the exposed partners, was strong evidence of a selective effect, and the differential deviation of the seroconverters, but not the persistently seronegatives, corroborated the difference. Neither chance nor systematic selection of couples into the study cohort by their *CCR2-CCR5 *profile unrelated to infection seems as plausible an explanation as the direct effect on acquisition of HIV-1 infection proposed here.

No *CCR2-CCR5 *variant carried by index partners was associated with an appreciable difference in transmission--not even the diplotype HHD/HHE associated with a statistically significant higher mean VL. This relative deviation in level of viremia was apparently not equivalent to the larger deviation conferred by index partner HLA-B*57, a genetic marker associated with a significantly lower transmission rate in this population [14]. Such differential impact of the different genetic markers may reflect a threshold effect by which a deviation of VL greater than a certain level overrides any genetic influence, but the number of subjects in our cohort was insufficient to assess that possibility.

We observed a trend toward an increased rate of acquisition among the exposed partners carrying HHF*2. In another African population (Cameroon), the frequency of CCR2-64I (HHF*2) was higher in the HIV-1 seropositives (most likely of mixed viral subtype) than in the seronegatives [30]. However, we remain skeptical about the importance of these findings for several reasons. First, the association and its significance in Zambians diminished in the multivariable analysis. Second, previous evidence for a role of HHF*2 in occurrence of infection is sparse, and there is no other report from a prospective study. Third, considerable uncertainty remains about the functional relevance of the HHF*2 polymorphism and *CCR2 *itself to HIV-1 infection [31]. Further population studies alone are unlikely to clarify more precisely the true nature of this genetic contribution.

More rapid HIV-1 acquisition among exposed seronegatives occurred in association with the HHD/HHE diplotype, and the association was stronger in exposed women than men. An association with this diplotype has not been reported before, most likely because the single SNP allele that distinguishes HHD from other haplotypes is only frequent enough in persons of African ancestry. The relatively higher frequency (7%) of HHD/HHE in our population than in Caucasians or other smaller groups of Africans may have facilitated detection of its effect. Associations with higher risk of mother-to-child transmission have been reported for HHD in Africans [32] and with homozygous HHD in African Americans [33]. HHE has also been reported to be detrimental for HIV infection as well as disease progression, but HHD/HHE has not been studied previously as a diplotype. Although our findings do not constitute exact replication of previous work, they appear to indicate consistent effects of the two haplotypes across populations with different viral subtypes.

The effects of HHD/HHE appeared stronger in male-to-female transmission. Differences in VL among the donor groups did not explain this difference according to direction of transmission. Nor did the difference arise from any obvious difference in age or sexual exposure of the two groups. For each subgroup stratified by gender, the number of seronegative subjects carrying these genotypes (diplotypes) was relatively small. Analysis based on larger samples will be necessary to reach a reliable conclusion about such gender-specific associations.

One feature of our study worth noting is the advantage of survival analysis of time to transmission/acquisition in detecting relationships that may be weaker in the cross-sectional or case-control approach often used to assess genetic influences on HIV-1 infection. Survival methods may be more sensitive in capturing time-dependent genetic effects on infection just as they have been in the analysis of disease progression.

We did not adjust statistically for the number of genetic polymorphisms tested. Rather we have emphasized those nominally significant associations with *CCR2-CCR5 *variants that have previously been implicated in HIV/AIDS and de-emphasized those whose involvement was less predictable from earlier studies. The previously documented HHE association with higher VL [11,17,28,29] provided ample rationale for interpreting our results as confirmatory without treating all haplotypes as equally likely to be involved. The impact of HHD/HHE on seroconversion was predicted somewhat less directly by earlier work associating HHD with a higher frequency of neonatal infection [33]. An even more important reason why these relationships cannot be readily dismissed as chance findings is that they were observed in the context of significant deviations from HWE of the haplotype distributions in each of the seropositive groups but not the seronegative group.

A consistent effect of the frequent HHE with higher VL in subtype C HIV-1-infected Africans as well as subtype B-infected Europeans and a stronger effect of HHD/HHE could have further ramifications. Since the response to antiretroviral treatment in Europeans may be modified by (Δ32) [34-36] and perhaps by other receptor variants [37,38], investigators in African settings should consider whether similar studies of *CCR2-CCR5 *polymorphism might provide epidemiologically or clinically useful prognostic information.

## Conclusions

In summary, our analysis of *CCR2-CCR5 *haplotypes consisting of common combinations of SNP alleles spanning those two genes has confirmed a previously reported association of haplotype HHF*2 with favorable response to HIV-1 infection; and our longitudinal analysis of seroconversion in HESN African heterosexual partners has detected probable contributions by the HHD/HHE diplotype to acquisition of infection [11,17,39]. Further insight into these relationships will be gained from studies of correlation between gene variation and gene function, as well as investigation of other representative and informative populations of infected and uninfected Africans.

## Methods

### Study population

Our study population comprised HIV-1 serodiscordant, cohabiting heterosexual couples enrolled in the Zambia-Emory HIV Research Project between 1995 and 2006. The procedures for screening, recruitment, counseling, follow-up visits and laboratory testing have been described elsewhere [15,40]. All couples whose HESN partner acquired virologically linked HIV-1 from the index partner during follow-up were included in this study. For closer comparability to the transmitters, nontransmission couples were selected from a large number based on self-reported behavioral or clinical measures of unprotected sex. Virologically linked HIV-1 transmission was defined as identity between viruses from index and seroconverting partners, according to phylogenetic analysis of sub-genomic sequences of *gag*, *env *(gp120 and gp41), and long terminal repeat regions [16,40]. Participant characteristics have previously been thoroughly examined as potential risk factors for transmission in this cohort [15,16,40,41]. Risk factors considered here include index partner (donor) viral load (VL), age of each partner, and genital ulceration/inflammation in each partner. The study population consisted of 567 couples with: a) adequate data and biologic material for both partners, b) observation of nontransmission couples for at least nine months, c) intra-couple virologic linkage when transmission occurred, and d) none of the partners on anti-retroviral treatment.

### Non-genetic factors

VL was quantified as the number of HIV-1 RNA copies per ml of plasma using Roche Amplicor 1.0 assay (Roche diagnostic Systems Inc., Branchburg, NJ) in a laboratory certified by the virology quality assurance program of the AIDS Clinical Trials Group (ACTG). The lower detection limit was 400 copies/mL of plasma. For this work, VL was transformed to log_10 _and treated as a continuous variable. Previous analyses [40] indicated that index partners with a medium number of HIV-1 RNA copies/mL (10^4^-10^5^, log_10 _= 4-5) or a high number of copies/mL (> 10^5^, log_10 _> 5) were more likely to transmit the virus than those with a low number (< 10^4^, log_10 _< 4).

### Genotyping

Genomic DNA was extracted from whole blood and buffy coats using the QIAamp blood kit and protocols recommended by the manufacturer (QIAGEN Inc., Valencia, CA). PCR-based typing differentiated the dimorphic variants at eight sites--one in *CCR2 *(the SNP encoding V64I--rs1799864) and seven in *CCR5 *[six SNPs in or adjacent to the cis-regulatory or promoter region (A29G--rs2856758, G303A--rs1799987, T627C--rs1799988, C630T--rs41469351, A676G--rs1800023 and C927T--rs1800024)] and the 32-bp deletion (Δ32--rs333). *CCR5 *haplotypes were typed by a combination of two methods: a PCR typing scheme and a TaqMan SNP typing scheme. The PCR typing scheme used 12 combinations of sequence-specific primers (SSP) plus four additional SSP reactions in conjunction with T627C-specific primers to define the A29G variant as described for previous work [11,13,17-20]. Combination of variants at the eight sites form nine relatively frequent *CCR2-CCR5 *haplotypes (HHA-HHE, HHF*1, HHF*2, HHG*1 and HHG*2) according to the nomenclature of the Tri-Service HIV-1 Natural History Study (TSS) [42]. HHF*2 is the only haplotype carrying the V64I mutation. A TaqMan genotyping assay was used to confirm the PCR-based SNP typing and assign *CCR5 *haplotypes for 126 individuals. TaqMan assays were performed using customized TaqMan probes for 7 SNP sites; SNP alleles were assigned after real-time PCR using the ABI 7500 Fast System (Applied Biosystems) according to procedures recommended by the manufacturer.

### Statistical analysis

Non-genetic factors (VL, age, gender, genital ulcer, genital inflammation, circumcision, and presence of sperm) were compared between seroconverting and non-seroconverting exposed partners using χ^2 ^and t-tests. Hardy-Weinberg equilibrium (HWE) for each SNP and CCR haplotype distribution was assessed using SAS Genetics (see below). HWE was calculated for the entire cohort and for four separate partner groups: transmission index, nontransmission index, seroconverting, and exposed uninfected partners. Associations of frequent haplotypes/genotypes with HIV-1 VL among the index partners and seroconverters were tested using general linear model (GLM) statistics with adjustment for age and gender.

For analysis of time-to-infection (transmission and acquisition), follow-up time for each couple was measured from the date of their enrollment into the cohort to 1) the date of HIV-1 infection (first seropositive visit) of the initially uninfected exposed partner or 2) the most recent seronegative visit prior to administrative censoring date (December 31, 2006). Time-to-infection was displayed in Kaplan-Meier plots, and comparisons between genetically distinctive groups were evaluated with Wilcoxon and log-rank tests. These plots illustrate differences in transmission associated with specific genetic markers; they do not reflect transmission rates in the entire prospectively observed discordant couple population. The overall annual HIV-1 seroincidence (7-8/100 PY) represents a one-half to two-thirds reduction in transmission following joint testing and counseling.

Statistical analysis of genetic variants of *CCR2 *and *CCR5 *consisted of testing hypotheses derived from earlier work on acquisition or progression of infection (See Additional File 1; Table S1) followed by systematic search for novel associations in our study population. Multivariable Cox proportional hazards models were used to control for non-genetic covariates. We estimated the hazard ratios (HR), its 95% confidence interval (CI), and the corresponding two-sided *P*-values. For hypotheses on genetic markers consistent with previously reported associations, statistical testing was performed without correction for multiple comparisons. All statistical analyses were done using SAS^® ^9.2 including SAS/Genetics™ (SAS Institute Inc., Cary, NC).

## List of Abbreviations

CCR5: C-C chemokine receptor 5; CCR2: C-C chemokine receptor 2; AIDS: acquired immunodeficiency syndrome; HIV: human immunodeficiency virus; HR: hazard ratio; VL: viral load; SSP: sequence-specific primers; SNP: single nucleotide polymorphism; GLM: general linear model; HWE: Hardy-Weinberg equilibrium; HHA, etc: human haplotype A, etc.

## Competing interests

The authors declare that they have no competing interests.

## Authors' contributions

RM* performed the statistical analyses and participated in the preparation of multiple drafts of the manuscript. LH* performed the laboratory work, participated in the statistical analyses and participated in the preparation of multiple drafts of the manuscript. WS assisted in planning the laboratory work, performed the assays, and reviewed the manuscript. IB prepared analytic data sets of the clinical, epidemiologic, and genetic data; and assisted in editing the manuscript. JM organized the cohort studies, supervised the data collection in the field and reviewed the manuscript. SA conceived the cohort studies, participated in the design of the genetic substudies, and reviewed the analyses and the manuscript. EH participated in the design of the genetic substudies, supervised the performance of the viral sequencing and viral load measurements, and reviewed the manuscript. SS participated in the analyses and in the editing of the manuscript. JT participated in the design of the genetic substudies, supervised all aspects of the genotyping, participated in the analyses and reviewed multiple drafts of the manuscript. RAK conceived the genetics studies, supervised the statistical analyses, and reviewed and edited all drafts of the manuscript.

## Supplementary Material

Additional file 1 **Table S1: Studies of associations between polymorphisms in *CCR2 *and *CCR5 *and acquisition or progression of HIV-1 infection**. Summary of the recent publications on *CCR2-CCR5 *haplotypes and association with HIV-1 acquisition or disease progression. Includes references [43-45] Click here for file

Additional file 2 **Table S2: *CCR2-CCR5 *haplotypes and diplotypes as observed in HIV-1 discordant Zambian couples**. Frequency of *CCR2-CCR5 *haplotypes and common diplotypes in overall Zambia cohort and subgroups. Rare diplotypes with count less than 12 in overall cohort are not shown. Click here for file
