# Supplementary material for: Association of chemokine receptor gene (CCR2-CCR5) haplotypes with acquisition and control of HIV-1 infection in Zambians
Source: Retrovirology. 2011 Mar 23;8:22. doi: 10.1186/1742-4690-8-22 (PMC3075214; doi:10.1186/1742-4690-8-22)
Supplement: Additional file 1 — Table S1: Studies of associations between polymorphisms in CCR2 and CCR5 and acquisition or progression of HIV-1 infection. Summary of the recent publications on CCR2-CCR5 haplotypes and association with HIV-1 acquisition or disease progression. Includes references [43-45] [file 1742-4690-8-22-S1.DOC]

**Additional File 1.**

**Table S1: Studies of associations between polymorphisms in *CCR2* and *CCR5*** and acquisition or progression of HIV-1 infection.

| **Marker/Combination or Haplotype** | **Acquisition/ susceptibility** | ***Ref** | **Progression** | ***Ref** |
| --- | --- | --- | --- | --- |
| HHA |  |  | + | [17] |
| HHA/HHA | - | [18] |  |  |
| HHA/HHF*1 | - | [18] |  |  |
| HHB | - | [43] |  |  |
| HHC | - |  | - | [17, 21] |
|  |  |  | + | [17] |
| HHC/HHC, HHC/HHD, HHC/HHE, HHB/HHC | - | [18] |  |  |
| HHD (or 59356-T) | - | [44] | - |  |
| HHD/HHE, HHD/HHG*1 | - | [18] |  |  |
| HHE | - | [42] | - | [13, 25] |
| HHE/HHE |  |  | - | [11, 17-18] |
| HHE/HHF2 | 0 | [42] |  |  |
| HHE/HHF*1, HHE/HHG*1, HHE/HHG*2 | - | [18] |  |  |
| HHF1 |  |  | - | [17] |
| HHF2 | 0 | [45] | 0 | [14, 20, 25, 31] |
|  | + | [42] | + | [11, 17, 19-20, 22] |
|  |  |  | - | [30] |
| HHF2/HHF2, HHF*2/HHG*1 | - | [18] |  |  |
| P1 (HHE, HHF1, HHF2, HHG1, HHG2) |  |  | - | [26, 28] |
| 59353-T (HHA, HHB, HHC and HHD) |  |  | + | [23] |
| 59029-G (HHA, HHB, HHC and HHD) |  |  | + | [10, 26] |
| 59029-G/G |  |  | + | [10] |
| 8 SNPs in 5'UTR | 0 | [45] |  |  |

* References include major published population studies of associations of *CCR5* SNPs or haplotypes with HIV transmission, acquisition, or disease progression.

+ Protective effect - delayed or lower HIV progression, transmission or AIDS development.

- Risk effect - accelerated or higher risk of HIV progression, transmission or AIDS development.

0 No significant effect observed.
