# Supplementary material for: Association of chemokine receptor gene (CCR2-CCR5) haplotypes with acquisition and control of HIV-1 infection in Zambians
Source: Retrovirology. 2011 Mar 23;8:22. doi: 10.1186/1742-4690-8-22 (PMC3075214; doi:10.1186/1742-4690-8-22)
Supplement: Additional file 2 — Table S2: CCR2-CCR5 haplotypes and diplotypes as observed in HIV-1 discordant Zambian couples. Frequency of CCR2-CCR5 haplotypes and common diplotypes in overall Zambia cohort and subgroups. Rare diplotypes with count less than 12 in overall cohort are not shown. [file 1742-4690-8-22-S2.DOC]

**Additional file 2:**

**Table S2: *CCR2-CCR5*** haplotypes and diplotypes as observed in HIV-1 discordant Zambian couples.

|  | Overall | | Index | | Non-Index | |
| --- | --- | --- | --- | --- | --- | --- |
| Haplotype | Count | Frequency | Count | Frequency | Count | Frequency |
| HHA | 601 | 0.265 | 305 | 0.269 | 296 | 0.261 |
| HHB | 44 | 0.019 | 25 | 0.022 | 19 | 0.017 |
| HHC | 178 | 0.079 | 69 | 0.061 | 109 | 0.096 |
| HHD | 370 | 0.163 | 190 | 0.168 | 180 | 0.159 |
| HHE | 310 | 0.137 | 138 | 0.122 | 172 | 0.152 |
| HHF*1 | 128 | 0.056 | 76 | 0.067 | 52 | 0.046 |
| HHF*2 | 480 | 0.212 | 259 | 0.228 | 221 | 0.195 |
| HHG*1 | 157 | 0.069 | 72 | 0.064 | 85 | 0.075 |
| Diplotype |  |  |  |  |  |  |
| HHA/HHA | 92 | 0.081 | 48 | 0.085 | 44 | 0.078 |
| HHA/HHC | 44 | 0.039 | 18 | 0.032 | 26 | 0.046 |
| HHA/HHD | 93 | 0.082 | 53 | 0.094 | 40 | 0.071 |
| HHA/HHE | 78 | 0.069 | 34 | 0.060 | 44 | 0.078 |
| HHA/HHF*1 | 26 | 0.023 | 16 | 0.028 | 10 | 0.018 |
| HHA/HHF*2 | 120 | 0.106 | 63 | 0.111 | 57 | 0.101 |
| HHA/HHG*1 | 36 | 0.032 | 15 | 0.027 | 21 | 0.037 |
| HHC/HHD | 30 | 0.027 | 11 | 0.019 | 19 | 0.034 |
| HHC/HHF*2 | 37 | 0.033 | 15 | 0.027 | 22 | 0.039 |
| HHD/HHD | 39 | 0.034 | 26 | 0.046 | 13 | 0.023 |
| HHD/HHE | 49 | 0.043 | 18 | 0.032 | 31 | 0.055 |
| HHD/HHF*2 | 78 | 0.069 | 37 | 0.065 | 41 | 0.072 |
| HHD/HHG*1 | 26 | 0.023 | 12 | 0.021 | 14 | 0.025 |
| HHE/HHF*2 | 78 | 0.069 | 44 | 0.078 | 7 | 0.012 |
| HHE/HHG*1 | 23 | 0.020 | 14 | 0.025 | 9 | 0.016 |
| HHF*1/HHF*1 | 13 | 0.012 | 10 | 0.018 | 3 | 0.005 |
| HHF*1/HHF*2 | 36 | 0.032 | 21 | 0.037 | 15 | 0.027 |
| HHF*2/HHF*2 | 45 | 0.040 | 27 | 0.048 | 18 | 0.032 |
| HHF*2/HHG*1 | 35 | 0.031 | 20 | 0.035 | 15 | 0.027 |
| Other^a^ | 156 | 0.138 | 65 | 0.115 | 118 | 0.208 |

^a^ Rare diplotypes observed in fewer than 12 individuals in the overall population are grouped together.
